# Probiotics for Parkinson’s Disease

**DOI:** 10.3390/ijms20174121

**Published:** 2019-08-23

**Authors:** Parisa Gazerani

**Affiliations:** Biomedicine Department of Health Science and Technology, Faculty of Medicine, Aalborg University, Frederik Bajers Vej 3B, 9220 Aalborg East, Denmark; gazerani@hst.aau.dk; Tel.: (+45)-9940-2412

**Keywords:** Parkinson’s disease, probiotics, prebiotics, synbiotics, gastrointestinal, gut, microbiota, dysbiosis

## Abstract

Parkinson’s disease (PD) is a complex neurological disorder classically characterized by impairments in motor system function associated with loss of dopaminergic neurons in the substantia nigra. After almost 200 years since the first description of PD by James Parkinson, unraveling the complexity of PD continues to evolve. It is now recognized that an interplay between genetic and environmental factors influences a diverse range of cellular processes, reflecting on other clinical features including non-motor symptoms. This has consequently highlighted the extensive value of early clinical diagnosis to reduce difficulties of later stage management of PD. Advancement in understanding of PD has made remarkable progress in introducing new tools and strategies such as stem cell therapy and deep brain stimulation. A link between alterations in gut microbiota and PD has also opened a new line. Evidence exists of a bidirectional pathway between the gastrointestinal tract and the central nervous system. Probiotics, prebiotics and synbiotics are being examined that might influence gut-brain axis by altering gut microbiota composition, enteric nervous system, and CNS. This review provides status on use of probiotics for PD. Limitations and future directions will also be addressed to promote further research considering use of probiotics for PD.

## 1. Introduction

Parkinson’s disease (PD) stands in second place after Alzheimer’s disease among common neurodegenerative disorders [1]. An overall incidence rate of 17 per 100,000 persons per year has been reported [2]. The onset of PD is usually at 65 years and older age, and it appears slightly more frequently in men than in women [2,3]. The aging of society around the globe is predicted to increase the population affected by PD and result in challenges for the provision of medical and socio-economic care [4]. PD typically is known to be associated with the progressive loss of dopaminergic neurons in substantia nigra pars compacta [5]. Neurons in this region and other brain regions also develop abnormal intracellular deposits known as Lewy bodies that contain aggregated α-synuclein [6]. Relationship between degeneration of dopaminergic neurons and α-synuclein aggregation is unknown; however, an elegant study [7] has proposed a possible mechanism underlying dopaminergic cell death and α-synuclein aggregation [8]. 

Motor impairment, characterized by resting tremor, muscle rigidity, and postural instability, has long been recognized as hallmarks of PD [9,10]. However, clinical features of PD include non-motor symptoms [11,12], including olfactory dysfunction [13,14], pain and sensory disturbances [15,16,17], and gastrointestinal (GI) dysfunction [18,19]. These symptoms have, in particular, been considered valuable for early diagnosis of PD [20] because they can occur years before initiation of motor symptoms [21]. A diverse range of biomarkers [22], from biological biomarkers (e.g., body fluids including cerebrospinal fluid, plasma, saliva, and recently tears [23,24,25,26]) to brain imaging biomarkers, have been introduced over years to assist better diagnosis and treatment of PD [5,22]. Considering PD complexity, a combination of multimodal biomarkers would be ideal to enhance diagnostic accuracy and personalized medicine for PD [22].

Besides levodopa, which is the most used medication for treating motor symptoms of PD, other therapeutic agents such as monoamine oxidase type B inhibitors, amantadine, anticholinergics, β-blockers, or dopamine agonists have also been used [27]. The rationale behind use of dopamine precursor and dopamine receptor agonists is to compensate for dopaminergic cell loss and to enhance dopaminergic load. It has been reported that levodopa was used by 85% of patients with PD in the USA (between 2001 and 2012), while dopamine agonists by 28% [28]. Levodopa, however, carries several side effects; it does not prevent dopaminergic neuron degeneration; it has no effects on non-motor symptoms [29]; prolonged use of levodopa can lead to levodopa-resistance [29] and levodopa induced dyskinesia [30], and PD-associated GI dysfunction contributes to levodopa response [31,32].

As a consequence of recent advances in the understanding of PD, a number of new therapeutic approaches have been introduced [33], including genetic and disease-modifying approaches to reduce abnormal accumulation and aggregation of α-synuclein, mitochondrial dysfunction, dysfunction of lysosomal proteins, blockade of neuroinflammation, and enhancement of neurorestoration [34]. Besides novel compounds and repositioned drugs, new technologies such as cellular therapies, immunotherapies, and vaccines have been emerged together with non-pharmacological approaches such as gene therapy, optogenetics, and deep brain stimulation, some of which already entered clinical phases of investigation [35]. A recent review has painted a bright future for PD within the next 20 years, where it can be slowed down, stopped, or reversed [36].

**Figure 1:**
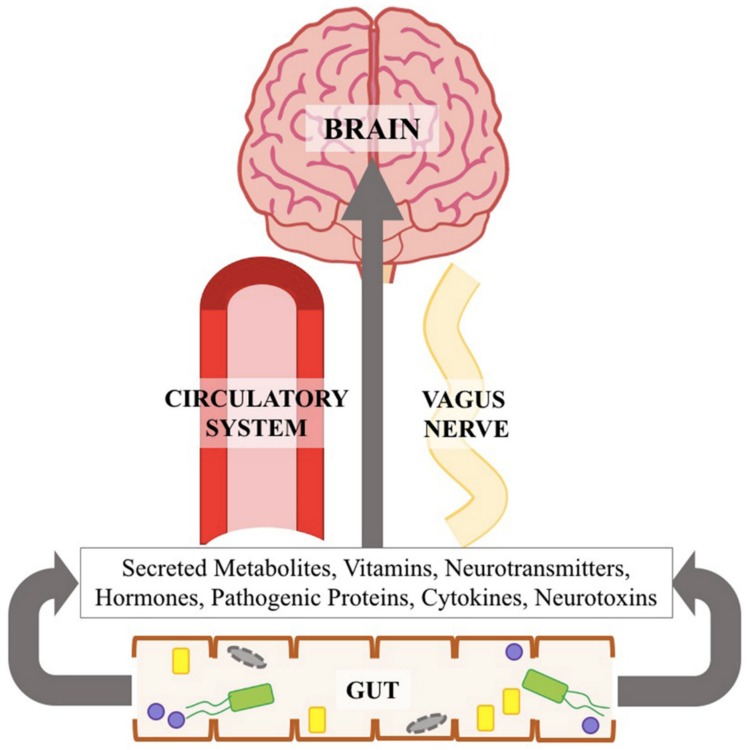
Communication between the gut microbiota and the brain [43] (reused with permission, license number: 4637571224608, Elsevier and Copyright Clearance Center).

Recently, GI tract, enteric nervous system (ENS) [37], gut microbiota, and a cross talk of gut-brain [19,38] have become a spot light as a potential mechanism underlying development of PD [39,40,41,42]. A simplified illustration of communication between gut microbiota and brain can be seen in Figure 1. 

GI dysfunction in PD was already highlighted by James Parkinson [44], and it is one of the most common non-motor symptoms with a prevalence of up to 80% [45]. GI dysfunction [18,46] appears as bloating, constipation, nausea, delayed gastric emptying, and prolonged intestinal transit time, leading to an incomplete defecation with a high negative impact on quality of life of patients with PD [45]. Constipation is highly prevalent (87%) and occurs before motor symptoms [47]. This may support the hypothesis that PD development and progression may occur at least in part with a contribution from the gut [39]. 

Aggregated α-synuclein has been found in neurons outside the CNS [48], including the ENS. This finding led Braak et al. [49] to hypothesize that α-synuclein aggregates are formed outside the brain and travelled from peripheral tissues. Vagal nerve has been proposed to provide the path for this spread from the ENS to brain [50,51]. Later studies [52,53] have suggested that aggregation can be triggered by gut microbiota and this process not necessarily needs a pathogen or an environmental toxin [54] as it formerly was suggested [50]. Several lines of evidence support that gut-brain axis is influenced by the gut microbiota [55,56,57]. 

**Figure 2:**
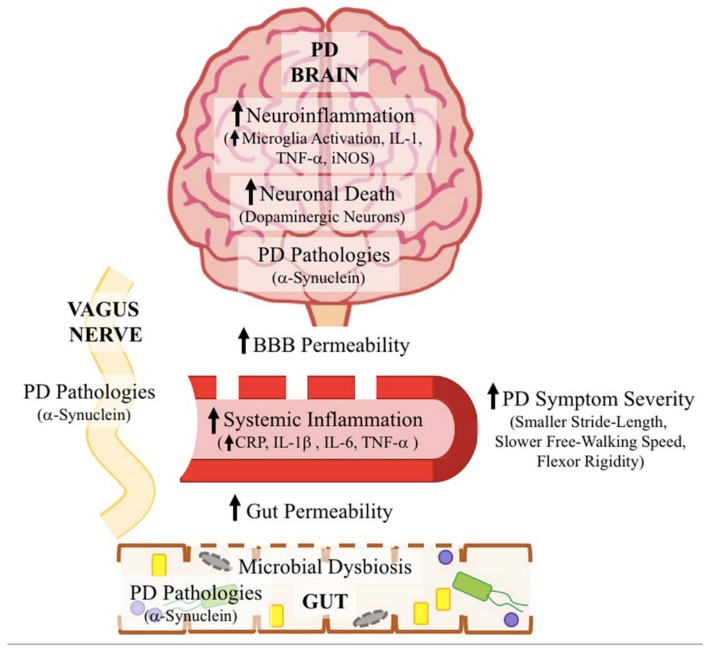
The role of gut microbiota in the pathogenesis of Parkinson’s disease. BBB, blood brain barrier; CRP, C-reactive protein; IL, interleukin; iNOS, inducible nitric oxide synthase; PD, Parkinson’s Disease; TNF, tumor necrosis factor [43] (reused with permission, license number: 4637571224608, Elsevier and Copyright Clearance Center).

Human GI tract hosts a diverse population of bacterial species, collectively called human gut microbiota, where a symbiosis exists between the host and bacteria [58,59,60]. Microbiome signatures in mouse, rat, non-human primate, and human faces have elegantly been studied and reported in 2018 [61]. Data from this study provide important information about host-specific microbiome signatures and can be used as a starting reference for future studies for investigation of gut microbiome in health and disease. Interestingly, findings from study [61] show several common genera between the studied species that are highly valuable for translational studies from preclinical phases to clinic. Considering some limitations in this study, further investigation has been recommended by the authors, including higher control of dietary factors and inclusion of both sexes [61]. This is due to the fact that gut microbiota is influenced by several factors, including sex hormones, and diet [61,62]. The latter is influenced by factors such as geographical location and ethnicity, accessibility, and habits. Therefore, future studies considering these factors are valuable. Maintenance of a healthy microbiota is important for gut barrier integrity, immunity, function, metabolism [62,63], and the gut-brain axis [64]. A potential role of this axis in several CNS-associated disorders has been highlighted, including in multiple sclerosis [65] and PD [66,67]. Alterations found in gut microbiota, including number and composition of gut microbiota and microbial metabolites [68], have been considered as valuable signatures for early diagnosis of several neurodegenerative disorders [43], including PD. Excellent recent reviews are available on alterations of gut microbiota and potential molecular mechanisms of gut microbiota linked to pathogenesis of PD (see Figure 2 for a schematic illustration), where, based on the literature, targeting microbiota with a diverse range of interventions has also been proposed [19,40,41,46,69,70,71,72,73,74,75,76,77,78]. This review briefly summarizes current knowledge on disturbed gut microbiota in PD, followed by status of targeted interventions, with a particular focus on the use of probiotics in PD.

## 2. Gut Dysbiosis in PD

A range of PD-associated GI dysfunctions has been clinically identified, including weight loss, gastroparesis, constipation, and defecation dysfunction [79,80,81]. GI dysfunction is reported a potential contributor to PD pathogenesis. This notion is supported by pathophysiologic evidence that α-synuclein inclusions appear early in the ENS, then reach the brain by e.g., vagal nerves [50,82]. A sub-type of intestinal epithelial cells called enteroendocrine cells provide the signaling pathway through which the microbiome interacts with the CNS via the vagus nerve [83,84]. It is worth mentioning that α-synuclein aggregates are also seen in the ENS of normally aging individuals [85]; however, they are more prevalent in patients with PD [86]. In addition, direct evidence exists that injection of human α-synuclein fibrils into the gut tissue of healthy rodents is sufficient to induce aggregated α-synuclein pathology within the vagus nerve and brainstem [87]. In a genetic mice model of overexpression of α-synuclein, the presence of gut microbiota has been demonstrated necessary to promote pathological alterations and motor dysfunction similar to what is seen in PD [88]. Interestingly, in the same mouse strain, fecal transplants from patients with PD could result in impairment of motor function, showing that gut microbiota is actively involved in initiation of α-synuclein proteinopathy in PD [88]. Once amyloids are produced by some members of the gut microbiota, they are released to extracellular space, and by neighboring cells including neurons, they can be internalized and form pathological aggregates of α-synuclein [89,90]. Clearance mechanisms such as the ubiquitin-proteasome system to degrade the misfolded protein are demonstrated failed in familial and idiopathic PD [91].

Alterations in gut microbiota–dysbiosis-is evident in PD [74,92]. Table 1 presents altered gut microbiota in patients with PD [74].

Patients with PD show an increased intestinal permeability correlated with intestinal α-synuclein accumulation [53]. It has been proposed that increased intestinal permeability and the translocation of bacteria and inflammatory bacterial products might lead to inflammation and oxidative stress in GI and thereby initiating α-synuclein accumulation in the ENS [53]. In addition, gut-derived inflammatory products can promote the disruption of the blood brain barrier and thus facilitate dopaminergic loss occurring in the SN [98,99]. In line with this hypothesis, biopsies of colonic tissue obtained from patients with PD have been found with an increased expression of proinflammatory cytokines, including TNF-α, IFN-γ, IL-6, IL-1β, and increased activation of enteric glial cells [100].

Reduction of *Prevotellaceae* in fecal samples of patients with PD was reported in 2015 [92]. Lower *Prevotellaceae* diminishes the levels of health-promoting neuroactive short chain fatty acids (SCFA) and the capacity for biosynthesis of thiamine and folate [101] that is seen decreased in patients with PD [101,102]. Interestingly, *Prevotella* decrease might be related to lower synthesis of mucin, which is associated with increased gut permeability [53]. In addition, lower *Prevotella* and higher presence of *Lactobacilliceae* have been associated with lower concentrations of ghrelin. Ghrelin is a gut hormone that may be involved in the maintenance and protection of normal nigrostriatal dopamine function [103]. In line with this, impaired ghrelin secretion has been reported in patients with PD [104]. Interestingly, hydrogen sulfide secreted by *Prevotella* has been shown to exert a protective effect on dopaminergic neurons in rat and mouse models of PD [105]. 

A study has reported that bacteria more commonly associated with anti-inflammatory properties, such as genus *Blautia*, *Coprococcus*, and *Roseburia*, are significantly reduced in fecal samples of patients with PD, along with a reduction in the genus *Faecalibacterium* and an increase in the genus *Ralstonia* in the GI mucosa of PD subjects, which potentially shifts the microbial balance within the colon to a more inflammatory phenotype [52]. 

Immune factors in stool of patients with PD have been profiled recently [106], and findings from this study indicated that intestinal inflammation was present in 156 individuals with PD compared with 110 controls. Elevated levels of vascular endothelial growth factor receptor 1, IL-1α, and CXCL8 were found in patients’ samples. Sex, body mass index, a history of smoking, and use of probiotics were found to strongly influence levels of stool analytes in this study [106]. Considering these factors in analysis, results demonstrated that elevated levels of IL-1α, CXCL8, IL-1β and C-reactive protein in the patients’ samples and results were not dependent on subject age or disease duration [106]. This study shows that measurement of selective immune factors in stool can facilitate identification of individuals at risk [106]. 

Stool samples of patients with PD compared with controls have also shown some alterations in fecal microbial composition and fecal SCFA concentrations [52,67]. SCFAs are important metabolic products of gut microbiota and exert central effects indirectly or directly on the ENS [107] and CNS [108]. Further metabolism of SCFAs results in Indican (indoxyl sulfate) that is eliminated in the urine, [109] and hence, urinary concentration of this substance can be considered a potential biomarker of gut dysbiosis [110]. 

Small intestinal bacterial overgrowth (SIBO) is also evident in patients with PD [78,111,112], and is associated with bloating, flatulence, and malabsorption, and more severe motor fluctuations, based on evaluation by a questionnaire with Global Symptomatic Score. SIBO might cause changes in intestinal permeability and contribute to an increase in bacterial translocation and therefore induce an inflammatory response [113]. The results of recent studies about gut microbiol dysbiosis in patients with PD have been summarized in an excellent review from 2018 [74]. 

A recent study showed significant differences in the gut phagobiota of patients with PD and a depletion of *Lactococcus* bacteria [114] in these patients that is associated with the regulation of gut permeability [115] and dopamine production [19], the two factors linked with the early gut signs of PD [116]. 

Dysbiosis in gut microbiota is not limited to human studies and has also been reported in animal models of PD. For example, a recent study revealed that decreased phylum *Firmicutes* and order *Clostridiales*, along with increased phylum *Proteobacteria*, order *Turicibacterales* and *Enterobacteriales*, were found in fecal samples of 1-methyl-4-phenyl- 1,2,3,6-tetrahydropyridine (MPTP)-induced PD mice, the increased abundance of *Proteobacteria* being consistent with observations in human subjects with PD [117]. Rotenone- treated mice have also exhibited fecal microbial dysbiosis characterized by an overall decrease in bacterial diversity and a significant change of microbiota composition, notably an increase in the *Firmicutes/Bacteroidetes* ratio after three weeks of rotenone treatment [118]. Sprague-Dawley rats treated with rotenone have also been shown with elevated *Bifidobacterium*, *Lactobacillus*, *Turicibacter,* and *Sutterella* in the small intestine and colon and decreased *Prevotella*, S24-7 and *Oscillospira* [119]. The results of recent studies about gut microbiol dysbiosis in animal models of PD have been summarized in an excellent recent review [74]. 

Taken together, evidence from human and animal studies demonstrates that alterations of gut microbiota exist in PD. Dysbiosis apparently results in differential production of a diverse range of substances in the gut that can influence gut-brain axis in PD. Various strategies have been used to study gut microbiota and how it might mediate PD pathology (e.g., germ-free, antibiotics, probiotics, fecal transplant, and infection). These strategies are summarized in a review paper from 2017 [76]. 

It is not possible to determine if changes in the gut microbiota are a cause or a consequence of PD pathogenesis, but the net result is the neuronal loss following inflammatory cascades or oxidative reactions [54]. Overall, gut-brain axis is a bi-directional communication pathway, and in relation to PD, this notion has increasingly been supported [19]. Subsequently, this has led to targeting the gut-brain axis in PD. 

## 3. Targeting Gut–Brain Axis in PD 

### 3.1. Antibiotics

One of the potential therapeutic possibilities is the application of antibiotics. These agents have been long known to alter gut microbiota. A current interest has been formed around additional effects of antibiotics (e.g., anti-inflammatory, anti-aggregating, and antioxidant properties) in neurodegenerative diseases [120,121,122], besides their ability to rebalance the gut dysbiosis [123]. Antibiotics, therefore, are likely to affect the gut microbiota-brain axis. For example, minocycline, presented neuroprotective effect in MPTP animal model, where it could cross the blood brain barrier and block dopamine loss [124]. It has been suggested that minocycline effect on gut-brain access is partly by modulating TLR4 [76]. Rifaximin has also been used for treatment of intestinal overgrowth, and this agent has also been reported to have an effect via TLR4 [125]. Further research is required to demonstrate that some antibiotics can act as alternative agents for PD. 

### 3.2. Fecal Microbiota Transplantation

Fecal microbiota transplantation (FMT) is a technique, where feces (the entire gut microbiota) from a healthy donor is delivered into the GI tract of a patient by re-establishing a gut microbial community [126]. FMT is considered a more comprehensive approach to restore the gut microbiota, and it has been used for treatment of GI infection or other disorders. For example, FMT is highly effective for recurrent *Clostridium difficile* infection [127], to the point that it has been approved by the United States Federal Drug Administration (FDA) [128]. However, one study has highlighted that FMT might have an impact on patients’ immune system [129]. FMT has successfully been applied for treatment of other GI disorders including inflammatory bowel disease and irritable bowel syndrome [130]. Non-GI diseases have also been investigated to find whether FMT can be beneficial, and this area includes CNS disorders [131] including autism and multiple sclerosis [132]. 

Animal studies in PD models have demonstrated that α-synuclein overexpressed mice receiving microbiota from patients with PD present higher physical impairments compared with transplants from healthy controls, highlighting that microbiome alterations can be a risk factor for PD [88]. Another study has presented that FMT protects MPTP-induced PD in mice by reducing the activation of microglia and astrocytes in the substantia nigra, and reducing expression of TLR4/TNF-α signaling pathway in gut and brain [117]. In this line, investigation of gut microbiota-miRNA interplay [133] has demonstrated that gut microbiota may affect host by producing miRNAs, and gut microbiota might be regulated by host-secreted miRNAs [134]. Gut microbiota modulates miRNA-associated mRNA expression patterns in the hippocampus of germ-free mice, where these transcriptional changes are sex-dependent [135]. This interesting study highlights a divergence between molecular pathways that control the gut-brain axis [135]. miRNAs control the TLR-signaling pathways, including regulation of TLR mRNA expression, receptor activation, binding to TLR or TLR-specific signaling, TLR-induced transcription factors, and cytokines [136]. Possible relationships between exosomes, miRNAs, and TLRs in the nervous systems have just started to be determined. However, hypothetically, miRNAs entering the cells via exosomes may tune the activation of TLRs [133], and that might be a useful strategy for modulation of the gut-brain axis in PD.

These findings highlight the value of further investigation via translational studies to assess if FMT approach would be beneficial for PD. A number of issues need to be considered for the use of FMT, including ethical issues, selection and screening of appropriate donors, risk and benefit assessment, emotional and behavioral consequences, and long-term safety [133,134].

### 3.3. Dietary Interventions

Dietary interventions [54] might influence the gut-brain axis by altering microbiota composition or by affecting neuronal functioning in the ENS and CNS [42,54,137]. Therefore, these interventions might provide opportunities to complement the traditional PD therapies [76]. Emerging evidence suggests that lifestyle factors can contribute to PD pathology. Smoking and coffee have been found associated with reduced risk of PD via their effects on gut microbiota [138]. There are number of excellent reviews on food-based treatments, dietary intervention, and probiotics for PD [54,139]. Below, the focus is on probiotics for PD. Table 2 presents the studies that have used microbial treatments for PD [72].

## 4. Probiotics, Prebiotics, Synbiotics 

It has been over 17 years since the scientific definition of probiotics was introduced with guidelines to ensure appropriate use of the term [144]. An expert panel defined probiotics as, “*live microorganisms which when administered in adequate amounts confer a health benefit on the host*” [145]. In 2014, a consensus panel made a small change replacing “which” with “that” [146]. Reid et al [144] have recently urged researchers to follow the precise definition and to stay consistent in order to help advance the development and validation of microbial therapies. Within this context, two other terminologies exist—prebiotics and synbiotics [147,148]. Prebiotics are mostly fibers that are non-digestible food ingredients and beneficially affect the host’s health by selectively stimulating the growth and/or activity of some genera of microorganisms in the colon, generally *lactobacilli* and *bifidobacteria* [147,148]. FAO/WHO defines prebiotics as “*a non-viable food component that confer health benefit(s) on the host associated with modulation of the microbiota*”. Synbiotics [149] are prebiotics combined with probiotics and this term is used for those products in which a prebiotic component selectively favors a probiotic microorganism [150]. 

Pro, pre, and synbiotics field offers a huge potential in science and marketing [144,147]. The common benefit of probiotics on gut microbiota is by restoring microbiota and maintaining immune homeostasis [151]. Probiotics have been shown to enhance intestinal epithelial integrity, protect gut barrier disruption, regulate immune system in GI mucosa, and inhibit pathogenic bacterial growth [152,153,154]. Most commonly used probiotic species and strains are presented in a recent review [155] including several *Lactobacillus spp.*, *Bifidobacterium spp.*, *Saccharomyces spp.*, and other coliform bacteria and their role in CNS disorders, obesity, diabetes, cancer, cardiovascular diseases, malignancy, liver disease, and multiple GI disorders to mention a few [155,156,157]. Probiotics have positive modulatory effects on brain function including reports showing normalization of anxiety [158,159] and depression-like behavior [160] through the gut-brain axis [139]. Long-term administration of the *Lactobacillus rhamnosus* strain in adult male BALB/c mice has been shown to reduce anxiety, depression, and stress responses, by modulation of central GABAAα2 but only in presence of intact vagus nerve. This finding highlights the importance of this communication path [161]. Our group has tested probiotic *Lactobacillus rhamnosus* PB01 on mechanical sensitivity and sperm kinematics in diet-induced obese mice [162,163]. An experimental study applying autoimmune encephalomyelitis model in mice has demonstrated that a mixture of probiotic *lactobacilli* works via another path than vagus nerve, and that is through induction of transferable tolerogenic regulatory T cells in mesenteric lymph nodes, in the periphery and CNS, which is mediated through a mechanism associated to IL-10 [164]. These examples show that effects and mechanisms underlying probiotics are diverse and broad spectrum and can be investigated through animal models of relevant diseases. Considering evidence in the literature in support of role of microbiota in various neurological disorders including PD [165], further attention and studies to investigate potential therapeutic use of probiotics in PD seem justified. Prebiotics and synbiotics have also been used but to a lesser extent. Probiotics are the most studied ones. Below, evidence available from these interventions for PD is provided. 

### 4.1. Probiotics for PD

Preclinical or clinical evidence on the beneficial effects of probiotics in PD is still very limited. There are potentials to predict why probiotics, prebiotics, and synbiotics might be beneficial in PD [54]. Probiotics might be a powerful tool in order to alter PD-associated microbiota composition, improve GI function, and therefore reduce gut leakiness, bacterial translocation and the associated neuro-inflammation in the ENS. The first clinical study was conducted in 2011 and demonstrated that patients with PD suffering from chronic constipation receiving fermented milk containing *Lactobacillus casei Shirota* for five weeks improved stool consistency and reduced bloating and abdominal pain [140]. This study highlighted the value of the used probiotic for improvements in stool consistency and defecation habits in patients with PD. Besides dairy products, non-dairy products in form of tablet have been shown capable of reducing abdominal pain and bloating in patients with PD in a study from 2016 [142]. This study used probiotics (60 mg per-tablet of two lactic bacteria: *Lactobacillus acidophilus* and *Bifidobacterium infantis*) for 3 months. 

A recent randomized, double blind, placebo-controlled clinical study [143] (ClinicalTrials.gov Identifier IRCT2017082434497N4) has looked into the effects of a probiotic product in capsule format (containing *Lactobacillus acidophilus*, *Bifidobacterium bifidum*, *Lactobacillus reuteri*, and *Lactobacillus fermentum*) on clinical (e.g., movement) and biochemical profiles (including metabolic parameters) in PD [143]. Administration of probiotic for 12 weeks resulted in favorable impacts on MDS-UPDRS (Movement Disorders Society-Unified Parkinson’s Disease Rating Scale), high-sensitivity C-reactive protein (hs-CRP), blood glutathione (GSH), malondialdehyde (MDA), and insulin metabolism but did not affect other metabolic parameters. In addition, this study demonstrated that probiotic consumption reduced insulin levels and insulin resistance and enhanced insulin sensitivity compared with placebo [143]. One major limitation of this study is that fecal bacteria loads were not determined prior and in response to the administered probiotics [143]. Authors have explained their findings in light of evidence in the literature for modulatory effects of probiotics on oxidative stress, insulin metabolism, and lipid profile [143]. 

Recently, a UK-led clinical trial [166] is due to begin to test if a probiotic drink could help improve both the motor and non-motor symptoms of PD. Researchers in this team have indicated that they use Symprove (a clinical trial of the probiotic Symprove (K-1803)), which is an oral drinkable probiotic that claims to be able to deliver live bacteria to the lower gut. This is based on the fact that bacteria in many commercial probiotics are unlikely to reach the lower gut, as most types of them, due to acidic environment in the stomach, are being inactivated before their final destination. Symprove is a multi-strain liquid probiotic that aims to get beneficial bacteria through the acidic stomach intact. This funded trial has been designed as a randomized, double blind, placebo-controlled trial and located in London. A total of 60 patients will be recruited and the intervention period is three months. 

Precise mechanisms underlying effects of probiotics in PD remain to be clarified. Most likely, effects are through multiple mechanisms. For example, improving gastrointestinal symptoms by probiotics can be a result of altering gut environment or inhibition of harmful gut bacteria [72]. For example, a lower abundance of *Prevotella* species in fecal samples from patients with PD has been reported [167] that can be corrected by probiotics. Another example is related to those patients with PD who are infected by *Helicobacter pylori*. It has been shown that these patients have lower absorption of L-DOPA [168]. Eradication of *Helicobacter pylori* by aid of some probiotics might then be useful in these patients. It has been shown that Probiotic *Bifidobacterium bifidum* CECT 7366 Strain affects *Helicobacter pylori* [169] and could be a potential option for future research in PD. *Lactobacillus reuteri* supplementation has also exerted anti *Helicobacter pylori* effects [170] and may offer some potentials for future investigation in PD. 

A lower count of *Bifidobacterium* species has also been found in the stool specimens from patients with progressive PD [171], proposing that probiotics might also be potentially useful in this case. 

Another potential mechanism is that probiotics can actually increase gut motility [172]. An in vitro organ bath study [173] has demonstrated that cell-free supernatants of *Escherichia coli* Nissle 1917 can directly stimulate intestinal smooth muscle cells. Findings from this study therefore suggest a potential mechanism of probiotics to modulate human colonic motility [173]. 

It has been shown that *Lactobacillus reuteri* accelerates gastric emptying and improves regurgitation in infants [174]. This might have an application for PD because delayed gastric emptying is common in patients with PD [175,176] and generally reduces absorption of PD medications including levodopa [32]. Identification of probiotics’ effects on gastric emptying and drug absorption in PD requires further investigation [72].

In general, consistency of stool has been found associated with gut microbiota richness and composition [177], and it seems justified to consider that probiotics could modulate constipation and or other GI motility issues [178].

Interestingly, there is in vitro evidence that probiotic bacterium *Bacillus* sp. JPJ can produce L-DOPA from Ltyrosine, which is then converted to dopamine with the aid of DOPA decarboxylase [179]. This study might have some translational potential for future studies. However, one should consider that biochemical synthesis of some of endogenous substances such as L-DOPA might not be useful alone and needs further clarification on how to be translated in vivo and the impact on pharmacodynamics and pharmacokinetics must be determined. 

There is another in vitro evidence from 2019 [180] that presents effects of probiotic (a selection of probiotics microorganisms belonging to the *lactobacillus* and *bifidobacterium* genus) in peripheral blood mononuclear cells (PBMCs) isolated from patients with PD compared to healthy controls. Investigators in this study assessed release of the major pro and anti-inflammatory cytokines, in addition to production of reactive oxygen species (ROS). In patients with PD, *L. salivarius* LS01 and *L. acidophilus* significantly reduced proinflammatory and increased the anti-inflammatory cytokines. LA02 resulted in ROS downregulation, remarkably in the early stages of disease. These findings emphasize that results might be disease-stage dependent. In addition, anti-inflammatory and antioxidant activities of LR06 and BS01 on PBMCs were different in cells obtained from males to females, which highlights a potential of sex-related response. Overall, findings from this study [180] pointed to promising results, but authors also acknowledged the limitations of the study including sample size and cross-sectional nature of the study design. Longitudinal in vivo studies to attempt reproduction of results have also been encouraged with an animal model of PD or direct evaluation of PD at clinic for both clinical and biological effects. 

Oxidative stress might be involved in apoptosis of dopaminergic neurons whether it is idiopathic or genetic cases of PD [123,181]. Probiotics that can promote production of antioxidant products, such as vitamins by gut microbiota, might offer beneficial effects for PD [76]. A number of vitamins have shown beneficial effects for patients with PD, including vitamin E, D3, Riboflavin, and vitamin B6 [76]. Healthy gut microbiota in humans produces vitamin K and most of the water-soluble B vitamins, such as biotin, cobalamin, folates, pantothenic acid, nicotinic acid, pyridoxine, riboflavin, and thiamine [182]. Probiotic strains such as *lactobacilli* and *bifidobacteria* are capable of producing potential antioxidants, vitamins, and bioactive molecules [183,184], potentially limiting free radicals load and exerting beneficial effects for disorders that are associated with oxidative stress including PD. 

Animal models of PD have shown that infiltrating CD4+ lymphocytes [185] and peripheral monocytes [186] into CNS contributes to neurodegeneration. Therefore, if probiotics exert immune regulatory effects, they might offer potentials for PD. It has been reported that oral *Pediococcus acidilactici* R037 could be effective in experimental autoimmune encephalomyelitis (EAE) [187]. *Lactobacillus plantarum* A7 and *Bifidobacterium animalis* have also been demonstrated effective in attenuating EAE progression in another study using this model [188]. In patients, probiotics have shown to modulate the microbiome and immunity in multiple sclerosis [189]. This study has tested oral probiotic containing *Lactobacillus*, *Bifidobacterium*, and *Streptococcus* twice-daily for two months and shown that the intervention resulted in anti-inflammatory response in the peripheral immune system characterized by decreased frequency of inflammatory monocytes, decreased CD80 on monocytes, and decreased human leukocyte antigen D on dendritic cells [189]. Examples presented above are not directly linked to PD but are based on the rationale that common mechanisms underlying disease progression have been shown to contribute in neurodegeneration caused by autoimmune responses and hence from this point of view might be useful to consider potential links to PD. There are currently no reports available to show if probiotics could exert an immune modulatory effect in PD; hence, this line is open for further investigation.

In addition, influence of SCFA on central microglia in PD and potential role of probiotics in this regard require further investigation [190]. There is evidence in literature to encourage more studies in this line. For example, in infants (three months age) with high risk of eczema, probiotic supplementation (*Bifidobacterium bifidum* W23, *Bifidobacterium animalis* subsp. *lactis* W52 and *Lactococcus lactis* W58, Ecologic(®)Panda) could modulate fecal SCFA [191]. Probiotic supplementation in this study resulted in higher levels of lactate and SCFAs and lower levels of lactose and succinate [191]. Another study in 2–5 year old children has also found beneficial effects of probiotic supplementation (*Lactobacillus paracasei* Lpc-37 or *Bifidobacterium lactis* HN019) for nine months on total *lactobacilli*, *bifidobacteria* and SCFAs [192], that could reduce risk for diarrhea and fever during the rainy season. 

Evidence on immunomodulatory role of probiotics in gut lipopolysaccharide (LPS) and its relation to PD is not known and can offer another line of investigation. Few studies are available to encourage further research. An in vitro study [193] has demonstrated that specific probiotics, in this case *B. longum* subsp. *infantis*, might decrease colonic LPS and consequently reduce the proinflammatory milieu [193]. An in vivo study has also shown that Malaysian LAB-fermented cow’s milk (CM-LAB), consisting of several *Lactobacilli*, could reduce LPS-induced neuroinflammation in mice and reverse memory deficits in this LPS-induced model [194]. 

Potential TLRs signaling has also been proposed that can be modulated by probiotics in relation to PD [133]. For example, *L. rhamnosus* (JB-1), *Lactobacillus casei* Shirota, and *Lactobacillus reuteri* by potential modulation of TLR1, TLR2, and TLR6 [140,161,195,196], 

It remains to be investigated whether and how probiotics can influence dementia and cognitive impairments in PD. There are some experimental and clinical data available for improvement of theses impairments for Alzheimer’s disease. For example, in a murine model of Alzheimer’s disease, *Bifidobacterium breve* strain A1 could reverse cognitive dysfunction [197]. Another mice model of Alzheimer’s disease also showed promising results in response to SLAB51 probiotic formulation, where the treated group showed reduction of cognitive decline compared with control [198]. Clinical data from a randomized, double blind, and controlled trial in 2016 show that 12 weeks of consumption of a mixed-species probiotic product in form of a milk, consisting of *Lactobacillus acidophilus*, *Lactobacillus casei*, *Bifidobacterium bifidum*, and *Lactobacillus fermentum* could improve mental state in patients with Alzheimer’s disease assessed by mini-mental state examination (MMSE) scores [199]. 

Use of probiotics for improvement in depression and anxiety in patients with PD is also very limited. In a germ-free mice model [200], administration of *Lactobacillus plantarum* PS128 was used to investigate emotional behaviors in response of gut-brain axis modulation. Results from this study presented an improvement in anxiety-like behaviors but not depressive behaviors, following the intervention [200]. This study proposes that behavioral responses (anxiety or depression) to probiotics might be different. Probiotics have also been tested for depression in humans and could show some promising results in irritable bowel syndrome [201] and in major depressive disorders [202]. *Bifidobacterium longum* NCC3001 reduced depression scores in irritable bowel syndrome [201], and *Lactobacillus helveticus* and *Bifidobacterium longum* in major depressive disorder [202]. According to these studies, probiotics might be useful for reducing depression or anxiety in patients with PD, but literature lacks any evidence so far. 

### 4.2. Prebiotics for PD

A range of prebiotics exists [147] that originate from different sources including soybeans, raw oats, unrefined wheat and barley, yacon, non-digestible carbohydrates, and non-digestible oligosaccharides [147]. A low-molecular-weight polysaccharide from agar and alginate of seaweed Gelidium CC2253 F, Ulvan from green algae-Ulvarigida, β-glucans from Pleurotus sp. (pleuran) mushrooms, inulin-type fructans from roots of traditional Chinese medicine Morindaofficinalis or Indian mulberry, oligosaccharide from white and red-flesh pitayas (dragonfruit), and oligosaccharide Yacon root have also been mentioned in the literature [147]. One must consider the prebiotic classification criteria and which one of these can fulfill the criteria to be included as prebiotics [54,203].

Prebiotic fibers have been shown to have beneficial effects on immune function, bowel motility, and constipation [54,204,205,206,207,208,209] that might be very relevant for inflammation and GI-related symptoms in PD. Moreover, prebiotics have been shown to increase the levels of brain-derived neurotrophic factor (BDNF) in the dentate gyrus of the hippocampus in rats [210]. BDNF signaling is critical for neuronal protection, survival, and plasticity [211]; therefore, supplementation might have implications on brain neuroprotection. In addition, fecal microbial community of PD has shown a lower abundance of SCFA butyrate-producing bacteria [52,67] that could be corrected by the use of prebiotic fibers.

### 4.3. Synbiotics for PD

Synbiotics exert beneficial effects on immune function, dysbiosis, and bowel function that collectively are relevant for PD. A clinical study has demonstrated that probiotic *Lactobacillus salivarius* decreased inflammatory markers in healthy subjects, and when it was combined with prebiotics, the effect was pronounced [212]. Another study has revealed that females with functional constipation that treated with a synbiotics yogurt, consisted of *Bifidobacterium animalis* combined with prebiotics, showed an increase in bowel movement, stool quantity, and quality compared to controls [213]. 

A study from 2016 (ClinicalTrials.gov identifier: NCT02459717) was based on a randomized, double blind, placebo-controlled trial in patients with PD with Rome III-confirmed constipation, in which daily intake of a fermented milk, containing multiple probiotic strains and prebiotic fiber, was tested for four weeks. The primary efficacy endpoint was the increase in the number of complete bowel movements (CBMs) per week and the findings presented constipation relief in patients compared to placebo [141]. Constipation severely affects the overall quality of life in patients with PD [214], and effective treatment options can offer great values at clinic. However, a link between dairy product consumption and increased risk of PD has been mentioned in the literature [215,216]. Potential adverse effects associated with the long-term use of fermented dairy products combined with probiotics are still unknown. In addition, we still do not know whether constipation relief following probiotics can also slow down progression of PD and to what extent. There is however a two-year follow-up study that examined gut dysbiosis correlation with progression of PD [171]. Therefore, future studies are encouraged to consider factors such as disease onset or progression into account while reporting quality of e.g., life of affected patients. 

The study by khalighi et al [217] showed that in patients with SIBO when treatment with antibiotics was followed by synbiotic supplementation containing *Bacillus coagulans* and prebiotics, a better response was obtained compared with antibiotics alone. The combined regimen also significantly decreased abdominal pain, flatulence, and diarrhea [217]. Since SIBO is prevalent in PD [78], and motor dysfunction is worse in these patients positive for SIBO [112], synbiotics have a potential to be considered for PD. 

Collectively, these studies listed above present potentials for probiotics, prebiotics, and synbiotics for PD. 

## 5. Conclusions and Future Perspectives

Accumulating evidence supports contribution of the gut-brain bidirectional pathway [19] and role of dysbiosis [74] in PD. A healthy gut microbiota can potentially decrease risk of developing several human disorders, most likely also PD [68]. α-synuclein deposition in PD might start in the ENS and propagate to CNS by trans-synaptic cell-to-cell transmission. Induction of a proinflammatory environment under dysbiosis conditions could also signal to brain through systemic pathways and dysfunctional blood brain barrier [54]. Consequently, an imbalance in host immune system might be responsible, at least in part, for motor and non-motor symptoms of PD. This new insight into PD pathogenesis has stimulated investigation of novel and early biomarkers and novel therapeutic strategies. Among options for correction of gut dysbiosis, probiotics, prebiotics, and synbiotics [148] have been investigated for effects on GI dysfunction, levodopa uptake, side effects of PD medications, and initiation or progression of neurodegenerative process. Findings from both preclinical and clinical data that were presented in this review have now proven that gut microbiota can directly or indirectly modify brain neurochemistry via various mechanisms including neural, immune, and endocrine processes. Based on this scientific rationale, further steps are expected in this field. This also shows that treatment of neurodegenerative disorders including PD may require a combination of therapeutic options, and those need to be individually adapted to disease process and progression for an optimal outcome [36,218].

Future studies on PD should account for the gut-brain axis and the manipulation of gut microbiota and microbial metabolites [68]. Even though recent evidence shows that gut microbiota can regulate CNS immune response, microglia, neurophysiological processes (e.g., neurotransmission), behavior, and blood brain barrier integrity, further understanding of mechanisms underlying these effects is highly encouraged. Cause-effect relationship between dysbiosis of gut microbiota and PD has been shown in particular in preclinical settings, and it has been shown that potentially a far more complex link exists beyond the unidirectional cause-effect relationship [68] and the fact that α-synuclein is now proposed to be a “bystander” contributing to multiple neurodegenerative processes, including PD [218]. Maintaining healthy gut microbiota may face limitations and challenges but the potential is currently promising. 

As presented here, probiotics, prebiotics, and synbiotics have demonstrated potential for PD; but when data are limited or mixed or weakness exists in quality of evidence, these interventions should not be exaggerated. We still need to find effects of exogenously administered probiotics on residential bacterial populations and intestinal microenvironments in patients with PD. There is also a need for more consistency in design of studies for probiotic strain types, strain combinations, duration of intervention, and application dosages. It is also important to define the “effect” of prebiotics, probiotics, or synbiotics clearly in study design to facilitate further interpretation of data and finding mechanistic information in relation to pathogenesis of PD. For example, inflammatory status and specific measures of inflammatory factors need to clearly facilitate our understanding in relation to impact on PD. Both preclinical and clinical studies would benefit from these factors because they can directly or indirectly influence trials’ outcomes. 

In future trials, composition of gut microbiota under optimal controlled conditions and after interventions needs to be assessed, and that might help individualized therapy in PD. Personalized probiotic approaches [219] might yield more reliable results, considering high variability in gut microbiota and the fact that effects of probiotics show high variability. To overcome some of those variabilities, use of genetically modified probiotics and new techniques for delivering these efficiently with high site specificity have been suggested [220]. A detailed analysis is crucial in determining which microbial communities are present as disease biomarkers in PD and also what are the potential responses to, e.g., probiotic intervention (considering most effective probiotics, dosage and duration) [192]. 

Identification of changes in PD microbiota that can regulate brain function remains a challenge. Multi-omics approaches can be employed including metagenomics, metaproteomics, and metabolomics [72]. An integrative analysis of multi-omics has not been done so far to investigate gut microbiota and their metabolites in patients before and after probiotic intervention. One study has identified microbiome signatures in several species including humans [61]. Programs such as the NIH Human Microbiome Project (HMP) could provide resources, methods, and discoveries on human microbiome and health-related outcomes [221]. This project has been carried out over ten years with two phases completed for three conditions of pregnancy and preterm birth, inflammatory bowel disease, and factors influencing individuals with prediabetes [222]. Genomic blueprint of human gut microbiota [223] is also available based on a recent study on 92,143 metagenome-assembled genomes obtained from 11,850 gut microbiomes [223]. These powerful tools are supposed to facilitate future research. 

It is also important to consider objective measures in addition to other self-report questionnaires like Rome III criteria for future trials of probiotics in PD. For example, colonic transit time is a valid indicator, and has been used to objectively evaluate the severity of constipation among patients with PD [224]. This concept of objective biomarkers can exploit to non-GI markers and those in PNS or CNS. For example, it has been proposed [218] that using wearable, non-invasive body worn devices can be useful to collect information on movement and daily activities in relation to disease progression and drug effects [225,226]. 

In future, correlational analysis to draw a link between two categories of findings, subjective and objective measures, are highly encouraged, for example, correlation between the stage of PD disease and outcome of probiotic intervention. At least, future studies are expected to report these parameters and factors that are taken into consideration for design and analysis. 

Another open question is whether persistent exposure to probiotics may lead to colonization of long-term residence in GI of patients with PD, or microbiota would return to its original after stopping probiotic intervention [72]. This would clarify whether continuous use of probiotics is required or can it be sufficient for a temporary period [72]. A longitudinal study, in which biomarkers and clinical findings can be collected before and after probiotics intervention, would likely provide important findings. Literature shows studies with single-strain probiotic and multispecies probiotics. Each strategy might have pros and cons, but one point is that identification of single strain makes some of the findings explanation more straightforward than those with multiple strains [72]. 

Last, but not least, there have been few studies addressing concerns about safety of probiotics [227], and future studies are encouraged to take safety biomarkers into account to be included in study design and reported beside efficacy measures. 

## Figures and Tables

**Table 1:** Altered gut microbiota compositions in PD patients [74] (reused with permission, license number: 4637580890170, Elsevier and Copyright Clearance Center).

Altered Microbiota	References
*Lactobacillus* ↑, *Clostridium coccoides* ↓, *Bacteroides fragilis* ↓,	[93]
*Prevotella (hydrogen sulfide producer)* ↓	
*Ralstonia* ↑, *Blautia* ↓, *Coprococcus* ↓, *Faecalibacterium* ↓, *Roseburia* ↓,	[52]
*Enterobacteriaceae* ↑, *Prevotellaceae* ↓	[92]
*Enterobacteriaceae* ↑, *Bifidobacterium* ↑, *Enterococcaceae* ↓,	[67]
*Lactobacillaceae* ↓, *Faecalibacterium prausnitzii* ↓, *Prevotellaceae* ↓	
*Verrucomicrobiaceae* ↑, *Firmicutes* ↑, *Erysipelotrichaceae* ↓,	[94,95]
*Prevotellaceae* ↓	
*Akkermansia* ↑, *Ruminococcaceae* ↑, *Lactobacillus*↑,	[66]
*Bifidobacterium* ↑, *Lachnospiraceae (SCFAs producer)*↓	
*Escherichia-Shigella* ↑, *Streptococcus* ↑, *Proteus* ↑, *Enterococcus* ↑, *Blautia (butyrate produce)* ↓, *Faecalibacterium (butyrate produce)* ↓, *Ruminococcus* ↓	[96]
*Clostridium IV* ↑, *Aquabacterium* ↑, *Holdemania* ↑, *Sphingomonas* ↑, *Clostridium XVIII* ↑, *Butyricicoccus* ↑, *Anaerotruncus* ↑, *Lactobacillus* ↓, *Sediminibacterium* ↓	[97]

**Table 2:** Comparison of studies using microbial treatment in Parkinson’s disease (PD) including a study with an animal model [72] (reused with permission, license number: 4637600311198, Springer Nature and Copyright Clearance Center).

Element	*N*	Type	Treatment Duration	Concentrations	Disease Model	Main Results	References
Probiotics	40	*Lactobacillus casei* Shirota (in fermented milk)	1× daily for 5 weeks	6.5 × 10^9^ CFU	PD patients	Improvements in stool consistency and defecation habits	[140]
Probiotic mixture with prebiotic fiber	80	*Streptococcus salivarius* subsp. *thermophilus*, *Enterococcus faecium*, *Lactobacillus rhamnosus GG*, *Lactobacillus acidophilus*, *Lactobacillus plantarum*, *Lactobacillus paracasei*, *Lactobacillus delbrueckii* subsp. *bulgaricus*, and *Bifidobacterium* (fermented milk)	1× daily for 4 weeks	2.5 × 10^11^ CFU	PD patients	Helped relieve constipation	[141]
Probiotics	20	*Lactobacillus acidophilus* and *Bifidobacterium infantis* (tablets)	2× daily for 12 weeks	120 mg/day Bacterial counts *	PD patients	Alleviated the symptoms of abdominal pain and bloating	[142]
Probiotics	30	*Lactobacillus acidophilus*, *Bifidobacterium bifidum*, *Lactobacillus reuteri*, and *Lactobacillus fermentum* (capsules)	1× daily for 12 weeks	8 × 10^9^ CFU/day	PD patients	Decrease MDS-UPDRS scores	[143]
FMT	15	Fecal flora from normal C57BL/6 mice	1× daily for 7 days	2 × 10^7^ CFU	MPTP murine PD model	Show neuroprotective effects on MPTP-treated PD mice by inhibiting glial cell activation and neuroinflammation	[117]

*N*, number of the intervention groups; *FMT*, fecal microbiota transplantation; *CFU*, colony-forming unit; *PD*, Parkinson’s disease; *MPTP*, 1-methyl-4-phenyl-1,2,3,6-tetrahydropyridine; *MDS-UPDRS*, the Movement Disorder Society-Unified Parkinson’s Disease Rating Scale; * CFU value was unavailable.
